# Gonadal transcriptomic analysis and differentially expressed genes in the testis and ovary of the Pacific white shrimp (*Litopenaeus vannamei*)

**DOI:** 10.1186/s12864-015-2219-4

**Published:** 2015-11-25

**Authors:** Jinxia Peng, Pinyuan Wei, Bin Zhang, Yongzhen Zhao, Digang Zeng, Xiuli Chen, Ming Li, Xiaohan Chen

**Affiliations:** Guangxi Key Laboratory of Aquatic Genetic Breeding and Healthy Aquaculture, Guangxi Academy of Fishery Sciences, 8th Qingshan Road, Nanning, 530021 China

**Keywords:** *Litopenaeus vannamei*, Gonad, Transcriptome, SSR

## Abstract

**Background:**

The Pacific white shrimp (*Litopenaeus vannamei*) is the world’s most prevalent cultured crustacean species. However, the supply of high-quality broodstocks is limited and baseline information related to its reproductive activity and molecular issues related to gonad development are scarce. In this study, we performed transcriptome sequencing on the gonads of adult male and female *L. vannamei* to identify sex-related genes.

**Results:**

A total of 25.16 gigabases (Gb) of sequences were generated from four *L. vannamei* gonadal tissue libraries. After quality control, 24.11 Gb of clean reads were selected from the gonadal libraries. *De-novo* assembly of all the clean reads generated a total of 65,218 unigenes with a mean size of 1021 bp and a N50 of 2000 bp. A search of all-unigene against Nr, SwissProt, KEGG, COG and NT databases resulted in 26,482, 23,062, 20,659, 11,935 and 14,626 annotations, respectively, providing a total of 30,304 annotated unigenes. Among annotated unigenes, 12,320 unigenes were assigned to gene ontology categories and 20,659 unigenes were mapped to 258 KEGG pathways. By comparing the ovary and testis libraries, 19,279 testicular up-regulated and 3,529 ovarian up-regulated unigenes were identified. Enrichment analysis of differentially expressed unigenes resulted in 1060 significantly enriched GO terms and 34 significantly enriched KEGG pathways. Nine ovary-specific, 6 testis-specific, 45 testicular up-regulated and 39 ovarian up-regulated unigenes were then confirmed by semi-quantitative PCR and quantitative real-time PCR. In addition, using all-unigenes as a reference, a total of 13,233 simple sequence repeats (SSRs) were identified in 10,411 unigene sequences.

**Conclusions:**

The present study depicts the first large-scale RNA sequencing of shrimp gonads. We have identified many important sex-related functional genes, GO terms and pathways, all of which will facilitate future research into the reproductive biology of shrimp. We expect that the SSRs detected in this study can then be used as genetic markers for germplasm evaluation of breeding and imported populations.

**Electronic supplementary material:**

The online version of this article (doi:10.1186/s12864-015-2219-4) contains supplementary material, which is available to authorized users.

## Background

The Pacific white shrimp (*Litopenaeus vannamei*) is a species of *Penaeus* shrimp that are native to the eastern Pacific Ocean, from the Mexican state of Sonora as far south as northern Peru [1]. It has become the world’s most prevalent cultured crustacean species as a result of its fast growth, adaptability to a wide range of salt and temperature, strong disease resistance, and low demand for dietary protein [2]. By 2004, global production of *L. vannamei* approached 1,116,000 tons, and exceeded that of *Penaeus monodon* [3]. By 2010, production had reached 2,721,000 tons [4]. However, the very limited supply of high-quality broodstocks is contrasted with the heavy demand of shrimp larvae from large-scale cultivation, especially in non-native countries such as China. The employment of inferior-quality broodstocks could lead to an eventual loss in gametic and larval quality, and production would then decline. Studies aimed at improving reproductive performance would therefore be helpful for the industrial applications of *L. vannamei*. However, previous studies primarily focused on disease resistance mechanisms and culturing techniques of *L. vannamei* [5–9], and baseline information related to its reproductive activity and molecular aspects of gonadal development remain scarce. Thus, it is important to understand the regulatory mechanisms of reproductive phenotypes in this species.

The first step toward understanding molecular mechanisms of reproduction is to identify and characterize sex-related genes and regulatory pathways. Many efforts have been made to reveal sex-related genes, and many of these genes are cloned and characterized in shrimp; for example, *M-phase phosphoprotein 6* (*MPP6*) [10], *cell division cycle 2* (*Cdc2*) [11], *cyclin A and cyclin B* [12], *gonad-inhibiting hormone* (*GIH*) [13], *mitogen-activating protein kinase 1* (*MAPK1*) [14], *prostaglandin reductase 1* [15], and *valosin-containing protein* (*VCP*) [16] in *Penaeus monodon*; and *sex-determiner transformer-2* (*Tra-2*) [17], *activated protein kinase C1* (*RACK1*) [18], and *cell apoptosis susceptibility* (*FcCAS*) [19] in Chinese shrimp *Fenneropenaeus chinensis*. In *L. vannamei*, *vasa-like*, *vitellin*, *gonadotropin-releasing hormone-like* and a sex-related marker have been identified [20–23]. Additionally, research methods, such as suppression subtractive hybridization SSH [24, 25], proteomic analysis [26, 27], EST sequencing [28–30], and microarray [28, 31, 32], have also been applied to scientific studies of shrimp in order to reveal potential sex-related genes. However, because of the lack of genomic sequences, comprehensive identification of sex-related genes and construction of regulatory networks associated with shrimp gonadal development are lacking.

Newly developed next-generation high-throughput sequencing technology has become a powerful tool for identifying genes involved in gonadal development, sex determination and sex differentiation [33–37]; and for SNP/SSR marker discovery [38–40] in aquaculture species where the genomic sequences are not available. In the present study, we performed transcriptomic sequencing of the gonads of 13-month-old adult male and female *L vannamei* to identify sex-related genes. The gonadal transcriptomic data of one-day post-eyestalk- ablation females (Day1O) and six-day post-eyestalk ablation females (Day6O) were also used for *de-novo* assembly and annotation so as to identify more genes during ovarian maturation. Results from the transcriptomic analysis would be particularly important for better understanding the regulation of gonadal development between sexes in this economically important aquaculture species. In addition, real-time PCR verification of 104 sex differentially expressed genes herein validates the reliability of the transcriptomic analysis strategy, and emphasizes some candidate genes of interest involved in sex determination and gonadal development for further functional studies.

## Methods

### Ethics statement

All procedures involving the handling and treatment of shrimp used in this study were conducted with the approval of the Animal Care and Use Committee of the Guangxi Academy of Fishery Sciences, Nanning, China.

### Experimental shrimp and sample collection

*L. vannamei* used in this study were reared at Fangchenggang aquaculture base, Guangxi Academy of Fishery Sciences, Nanning, China. The experimental shrimp were 13 months of age with a weight of 40–50 g. First, shrimp were anesthetized on ice for three minutes, then testes from male shrimp (Testis), and ovaries from the pre-eyestalk ablation female shrimp (PreO) were removed. The ovaries of one-day post-eyestalk ablation female shrimp (Day1O) and the six-day post-eyestalk ablation female shrimp (Day6O) were also isolated. The gonadal tissues (testis for male and ovary for female) were cut into cubes of approximately 0.5 × 0.5 × 0.5 cm in size and immediately immersed in liquid nitrogen overnight, and then stored at −80 °C until RNA extraction. Some pieces of gonad from each shrimp were fixed in 4 % PFA and sectioned at 5 μm for hematoxylin and eosin (HE) staining and observation by light microscopy.

### RNA isolation, library preparation, and Illumina Hiseq2500 sequencing

Total RNA was isolated using TRIzol® Reagent (Invitrogen, CA, U.S.) according to the manufacturer’s instructions, and genomic DNA was removed using DNase I (Takara, Tokyo, Japan). Then RNA quality was determined with a 2100 Bioanalyser (Agilent, CA, U.S.) and quantified using a ND-2000 (NanoDrop Technologies, DE, U.S.). Three RNA samples from each group (Testis, PreO, Day1O, and Day6O) were pooled equally, and the mRNA-seq libraries were then prepared using 5 μg of pooled total RNA and the TruSeqTM RNA sample preparation Kit (Illumina, San Diego, CA). First, mRNA was isolated with oligo(dT) beads and then fragmented to 100–400 bp by fragmentation buffer. Second, double-stranded cDNA was synthesized using a SuperScript double-stranded cDNA synthesis kit (Invitrogen, CA) with random hexamer primers (Illumina). Then the synthesized cDNA was subjected to end-repair, phosphorylation, and ‘A’ base addition according to Illumina’s library construction protocol. The libraries were amplified by PCR for 15 cycles using Phusion DNA polymerase (NEB), and then target cDNA fragments of 200–300 bp were selected using 2 % Low Range Ultra Agarose gel. After quantification by TBS380, paired-end sequencing of 101 bp reads was performed for the four cDNA libraries in one lane on an Illumina HiSeq2500 high-throughput sequencer.

### De-novo assembly and annotation

The raw paired-end reads were trimmed and quality controlled by filter fq (BGI internal software) to remove reads with adaptors, reads with unknown nucleotides larger than 5 % and low-quality reads (the rate of reads in which the quality value ≦ 10 was more than 20 %). Then the clean data from samples (Testis, PreO, Day1O, Day6O) were used to perform *de-novo* assembly with Trinity (https://github.com/trinityrnaseq/trinityrnaseq/wiki; version Trinityrnaseq_r2013-02-25) and with min_kmer_cov set to 2 and all other parameters set to default [41]. The longest- assembled sequences per gene model were called contigs. Then the reads were mapped back to the contigs, as with paired-end reads we are able to detect contigs from the same transcript as well as the distances between these contigs. Finally, we retrieved sequences without Ns and these could not be extended at either end. Such sequences were thereby defined as Unigenes. TGICL (http://sourceforge.net/projects/tgicl/files/tgicl%20v2.1/; v2.1) [42] was used to further assemble all the unigenes from different samples to form a single set of non-redundant unigenes (called all-unigenes). The all-unigenes displaying >70 % sequence identities were grouped into a cluster, in which the prefix is CL, and the cluster ID is locater after. And the others were singletons whose the prefix was Unigene.

The completeness of the transcriptome assembly was tested by Core Eukaryotic Genes Mapping Approach (CEGMA) software (cegma_v2.4.010312, using the default parameters), by comparing known 248 Core Eukaryotic Genes (CEGs) and the transcripts assembled by Trinity. CEGMA was developed to identify a subset of 248 highly conserved core eukaryotic genes (CEGs) deriving from six diverse model organisms in eukaryotic genomes [43].

All non-redundant unigene sequences were searched against protein databases (Nr, SwissProt, KEGG, COG.) using blastx (evalue < 0.00001) and the nucleotide database NT (e-value < 0.00001) by blastn (evalue < 0.00001) (http://blast.ncbi.nlm.nih.gov/Blast.cgi; v2.2.26 + x64-linux). Protein function information was predicted from annotation of the most similar protein available in the databases.

BLAST2GO (http://www.blast2go.com/b2ghome; v2.5.0, release 2012-08-01) [44] program was used to retrieve GO annotations of unigenes for describing biological processes, molecular functions and cellular components. Metabolic pathway analysis was performed using online KEGG Automatic Annotation Server (http://www.genome.jp/; BGI internal version, Release 63.0) [45].

### Identification of sex-specifically expressed and differentially expressed genes

SOAPaligner/soap2 (http://soap.genomics.org.cn/) was used to map the reads to the assembled transcriptome. Unique mapped reads including paired-end reads for which only one part matched, were used to calculate the level of gene expression using the fragments per kb per million fragments method (FPKM) [46]. The method edgeR was used to identify differentially expressed genes (DEGs) between two samples [47]. The threshold for the P-value was determined by the false-discovery rate (FDR). Unigenes with FDR ≤ 0.001 and ratio of FPKMs of the two samples larger than 2 (genes for which FPKM < 1 were filtered) were considered to be differentially expressed genes in this study.

In addition, functional-enrichment analysis was performed to identify which GO terms and metabolic pathways were significantly enriched in DEGs. Hypergenometric test and 0.05 cutoff P adjusted were used for analysis of the enrichment of functional terms. GO functional enrichment and KEGG pathway analysis were carried out using Goatools (https://github.com/tanghaibao/Goatools) and KOBAS (http://kobas.cbi.pku.edu.cn/home.do) [48].

### Simple sequence repeat (SSR) detection

SSRs were detected among the unigenes using MISA software (MIcroSAtellite; http://pgrc.ipk-gatersleben.de/misa/misa.html; version 1.0). Six types of SSRs were investigated: mono-, di-, tri-, quad-, penta-, and hexa-nucleotide repeats; and we kept the SSRs in which the lengths at both ends of the Unigene were more than 150 bp for primer design by Primer 3 (http://www.onlinedown.net/soft/51549.htm; Release 2.3.4).

### Semi-quantitative and quantitative real-time PCR validation

Quantitative and semi-quantitative real-time PCR were used to verify sex differentially expressed genes as identified from the gonadal transcriptome.

The sequences of 47 up-regulated testicular and 57 up-regulated ovarian unigenes were chosen for primer design using Array Designer 4 (http://premierbiosoft.com/dnamicroarray/index.html). The *β-actin* (BQF: 5′-GTGTGACGACGAAGTAGC-3′, BQR: 5′-GATACCTCGCTTGCTCTG-3′) was used as a reference gene. Total RNAs were reverse-transcribed with Goscript™ reverse transcription system (Promega, U.S.) according to the manufacturer’s instructions.

Primers for Testis-specific (FPKM of PreO = 0 and FDR ≦ 0.001) and PerO-specific (FPKM of Testis = 0 and FDR ≦ 0.001) unigenes were first amplified by semi-quantitative PCR using equivalent cDNAs from testis and ovary of pre-eyestalk ablation shrimp as templates. PCR products were analyzed with gel electrophoresis using 1.2 % agarose gel. When a gene was found to be expressed only in testis, the gene was considered to be a testis-specific gene, while a gene that was expressed only in PreO was considered to be an ovary-specific gene. For the genes that were expressed in both ovary and testis, quantitative real-time PCR was further conducted to verify whether they were differentially expressed in ovaries and testes. The primers for other DEGs were also verified by quantitative real-time PCR.

The quantitative real-time PCR was performed in the Applied Biosystems 7500 fast real-time system using THUNDERBIRD qPCR Mix (TOYOBO, Japan) as recommended by the manufacturer. The original cDNAs were diluted 100-fold for the target gene and *β-actin* amplification, and the PCR cycle for both the target genes and the *β-actin* was as follows: 3 min at 94 °C; then 36 cycles of 15 s at 94 °C, 30 s at 56 °C and 30 s at 72 °C; followed by 10 min at 72 °C. The PCRs used to detect all the target genes and *β-actin* reference gene were performed with three biologic replicates. The specificity of the amplification was assessed by a melting curve analysis to exclude primers with nonspecific amplification peaks. The relative expression level of target genes was calculated with the 2^−ΔΔCT^ method [49]. The Student’s *t*-test was conducted using SPSS 23.0 (http://www-01.ibm.com/software/analytics/spss/), and significant differences were determined at a *P*-value < 0.05 (two-tailed test).

## Results

### Sequence analysis and assembly

**Fig. 1:**
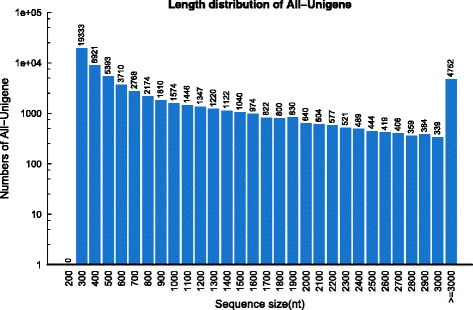
Length distribution of all-unigenes. X-axis, size distribution of unigenes; Y-axis, number of unigenes in different length ranges

A total of 25.16 gigabases (Gb) of sequences were generated from four *L. vannamei* gonadal tissue libraries (The raw reads data can be obtained from the NCBI Short Read Archive [SRA] under accession number SRA SRP059164.) After quality control with filter_fq, 63,782,344, 60,300,542, 58,195,380, and 61,048,948 clean reads were retrieved from the testis, PreO, Day1O and Day6O library, respectively. *De-novo* assembly of all the clean reads from four libraries generated a total of 65,218 unigenes with a mean size of 1021 bp and N50 of 2,000 bp (Table 1) (The assembled transcriptome can be obtained from the NCBI Transcriptome Shotgun Assembly (TSA) Database under accession number GDUV00000000). The length distribution of the unigenes obtained is illustrated in Fig. 1. The completeness of the transcriptome assembly was tested by CEGMA software by comparing known 248 Core Eukaryotic Genes (CEGs) and the transcripts assembled by Trinity. As a result, 234 out of 248 (94.35 %) CEGs were deemed to be complete proteins in the transcriptome, and 245 out of 248 (98.79 %) CEGs were found in the transcriptome including some partial proteins (Table 2), indicates a high level of completeness of the transcriptome.

**Table 1:** Summary statistics of the gonadal transcriptome of *L. vannamei*

Samples	Testis	PreO	Day1O	Day6O	Total
Total number of raw reads	64,971,684	61,702,958	59,778,572	62,654,322	249,107,536
Overall length of raw reads (Mb)	6562.14	6232.00	6037.64	6328.09	25159.86
Total number of clean reads	63,782,344	60,300,542	58,195,380	61,048,948	243,327,214
Overall length of clean reads (Mb)	6314.30	5981.34	5768.63	6049.23	24113.50
Number of unigenes	/	/	/	/	65,218
Mean length of unigenes (bp)	/	/	/	/	1,021
N50 length of unigenes (bp)	/	/	/	/	2000

**Table 2:** Statistics regarding completeness of the Unigene assembly based on 248 CEGs

	Complete					Partial				
	Prots	Completeness(%)	Total	Average	Ortho(%)	Prots	Completeness(%)	Total	Average	Ortho(%)
Group1	60	90.91	138	2.3	65	64	96.97	155	2.42	65.62
Group2	52	92.86	110	2.12	59.62	55	98.21	128	2.33	67.27
Group3	60	98.36	127	2.12	51.67	61	100	138	2.26	57.38
Group4	62	95.38	113	1.82	38.71	65	100	122	1.88	41.54
All	234	94.35	488	2.09	53.42	245	98.79	543	2.22	57.55

Group: Set of genes selected by Genis Parra; Prots: Number of 248 ultra-conserved CEGs present in the transcriptome; %Completeness: Percentage of 248 ultra-conserved CEGs present; Total = total number of CEGs present including putative orthologs; Average = average number of orthologs per CEG; % Ortho = percentage of detected CEGS that have more than 1 ortholog. ‘Complete’ refers to those predicted proteins in the set of 248 CEGs that when aligned to the transcriptome, give an alignment length that is 70 % of the protein length. If a protein was not complete but still exceeded a pre-computed minimal alignment score, then we called the protein ‘partial’. A protein that was deemed to be ‘Complete’ was then also included in the set of Partial matches

### Sequence annotation

All-unigene sequences were searched against Nr, SwissProt, KEGG, COG and NT databases, which returned 26,482 (40.61), 23,062 (35.36), 20,659 (31.68), 11,935 (18.30), and 14,626 (22.43 %) matches, respectively; providing a final total of 30,304 annotated unigenes (46.47 %) (Table 3).

**Table 3:** Statistics of annotation results

Databases	NR	NT	Swiss-prot	KEGG	COG	GO	All
Annotated unigenes	26,482	14,626	23,062	20,659	11,935	12,320	30,304

The 20,659 unigenes with a KO annotation were mapped to 258 pathways (Additional file 1: Table S1). The top 3 pathways were metabolic pathways (1,517 unigenes), RNA transport (562 unigenes), and regulation of actin cytoskeleton (501 unigenes). Importantly, the main biological pathways involved in germ cell meiosis during gonadal development were cell cycle (ko04110, 233 unigenes), DNA replication (ko03030, 61 unigenes), mismatch repair (ko03430, 41 unigenes), base excision repair (ko03410, 73 unigenes), oocyte meiosis (ko04114, 141 unigenes) and homologous recombination (ko03440, 41). The primary pathways involved in oogenesis, spermatogenesis and gonadal maturation were MAPK signaling pathway (ko04010, 309 unigenes), GnRH signaling pathway (ko04912, 118 unigenes), progesterone-mediated oocyte maturation (ko04914, 153 unigenes), focal adhesion (ko04510, 412 unigenes), calcium signaling pathway (ko04020, 229 unigenes), ubiquitin mediated proteolysis (ko04120, 344 unigenes) and wnt signaling pathway (ko04310, 228 unigenes). Further investigation of these pathways would be expected to reveal the regulatory mechanisms governing reproductive processes in *L. vannamei*.

### Sex-biased gene identification and enrichment analysis

**Fig. 2:**
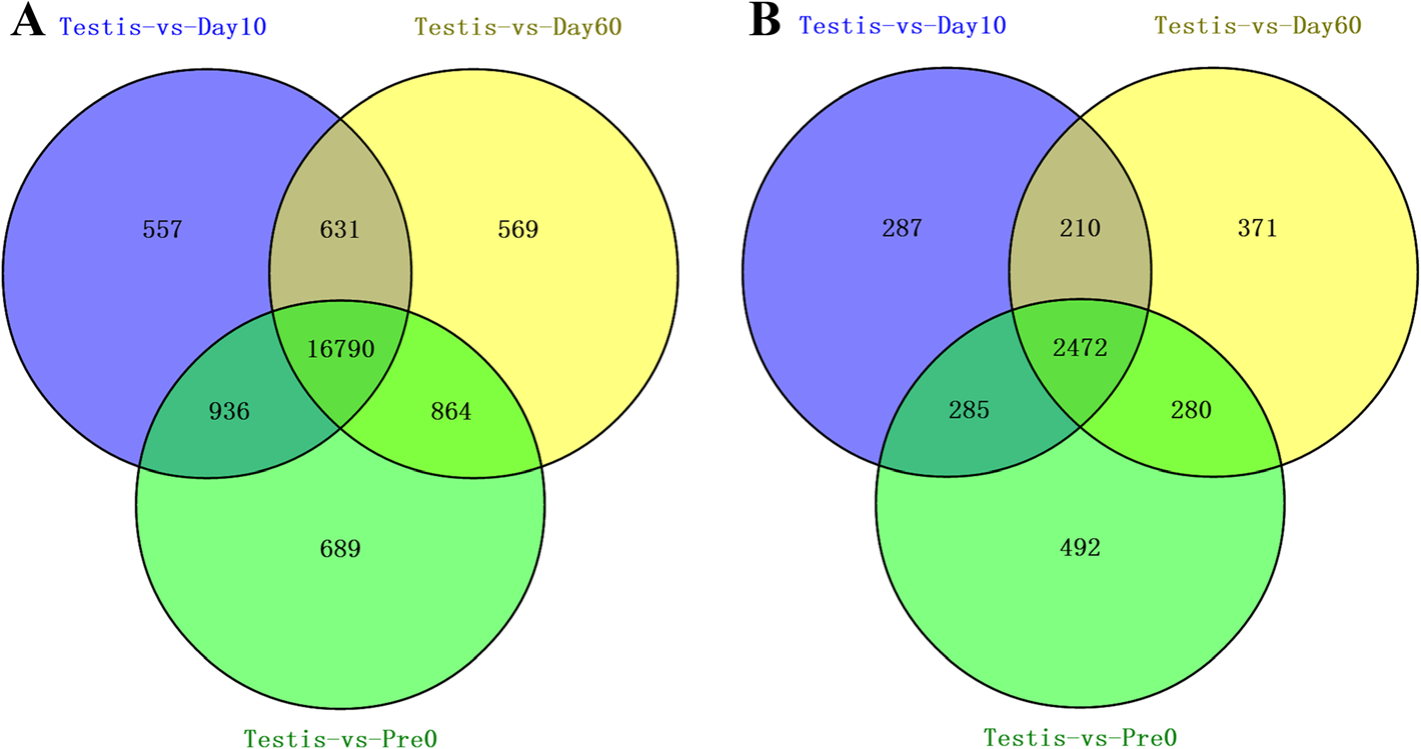
Statistical evaluation of differentially expressed genes between testis and ovaries that were obtained at different points in time. **a**: Venn diagram of DEGs that showed more expression in the testis than in the ovaries; **b**: Venn diagram of DEGs that showed more expression in the ovaries than in the testis

Comparison of gene expression levels in testis and different ovarian stages revealed similar numbers of differentially expressed genes (testis vs. PreO, 22,808 DEGs; testis vs. Day1O, 22,168 DEGs; testis vs. Day6O, 22187 DEGs), and over 84 % of the DEGs between testis and different ovarian stages are always the same (Fig. 2); and unigenes of testis and PreO libraries were selected for further analysis. Among 45,998 unigenes expressed in testis and ovary with lengths > 200 bp and FPKM > 1, 22,808 (45.6 %) showed sex-biased expression (Additional file 1: Table S2); 3,529 unigenes were up-regulated in the ovary and 19,279 were up-regulated in the testis (Fig. 2). Interestingly, the number of male-biased genes was much greater than that for female-biased genes, and the testicular up-regulated genes showed a greater average in fold-change than did the female-biased transcripts (Calculating from data in Additional file 1: Table S2, the average FC for male-biased and female-biased genes was 106.9 and 21.1, respectively).

**Fig. 3:**
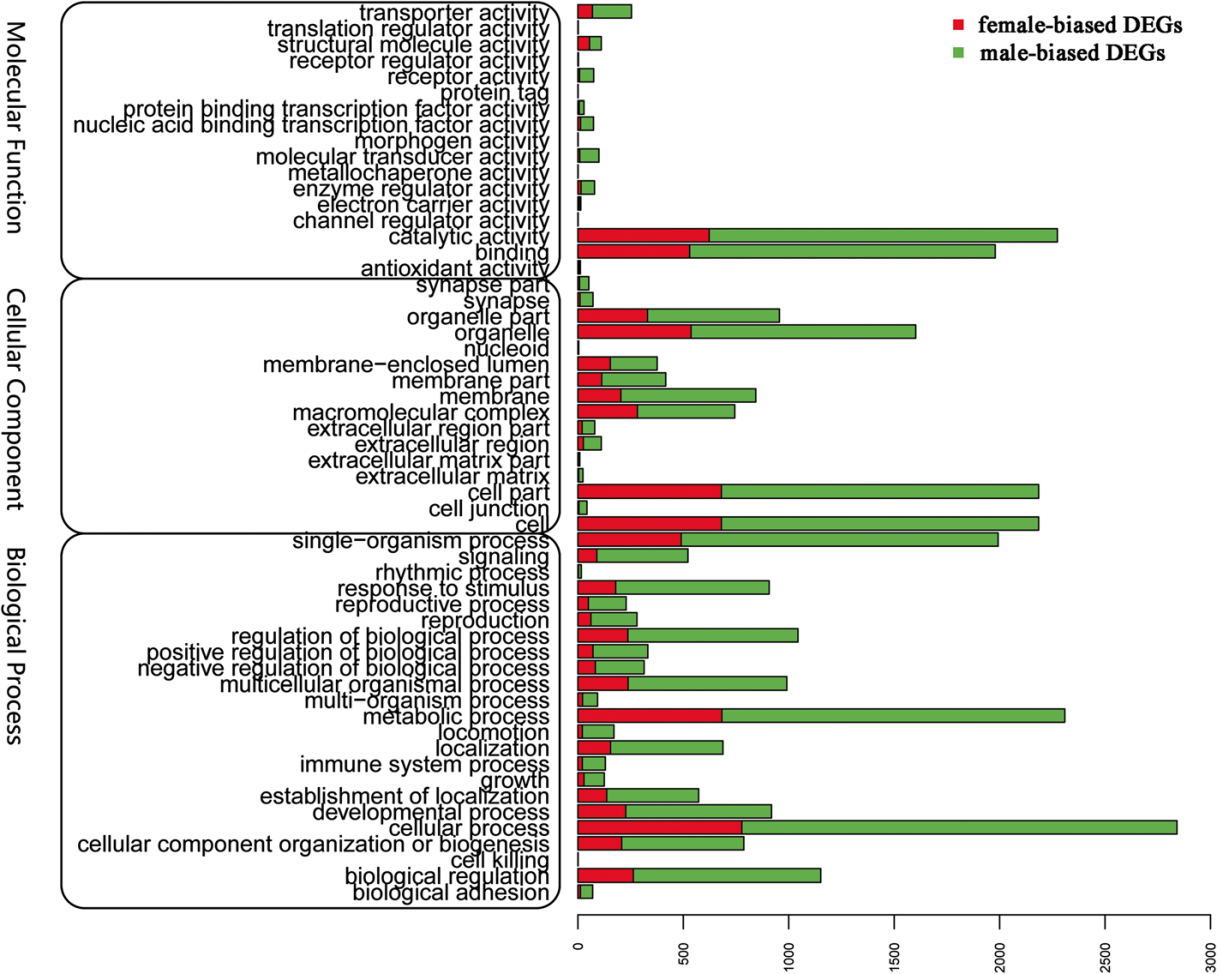
Distribution of DEGs among GO terms in biological processes, molecular functions, and cellular components

Gene ontology (GO) annotation was performed to classify sex-biased genes (Fig. 3). Results also showed a much higher number of male-biased genes than female-biased genes. Most of the GO terms showed significantly higher counts for male-biased genes. At the molecular function level, there were significantly more DEGs in catalytic activity and binding GO terms than in other terms. The cell part and cell GO terms had greatest DEG counts at the cellular component level. At the biological process level, cellular processes, metabolic processes, and single-organism processes had the top counts. By enrichment analysis, there were 49, 53, and 312 enriched GO-terms for male-biased DEGs, and 111, 155, and 380 enriched GO-terms for female-biased DEGs, in the cellular component, the molecular function and the biology process categories, respectively (Additional file 1: Table S3). The most enriched GO-terms for male-biased DEGs included DNA-directed RNA polymerase in the cellular component category, RNA polymerase activity in the molecular function category and isoprenoid biosynthetic and metabolic process in the biology process category (Table 4). As for female-biased DEGs, the most enriched GO-terms at cellular component level was mitochondrion and ribosome, the most enriched GO-terms associated with molecular function were aminoacyl-tRNA ligase activity, and the most enriched biology process were translation, cellular biosynthetic process and organic substance biosynthetic process (Table 4).

**Table 4:** Representative enriched GO terms (Bonferroni-corrected P-value ≤ 0.05) and pathways for sex-biased (Q value ≤0.05) genes

Enriched GO terms for sex-differentially expressed genes				Enriched pathways for sex-differentially expressed genes			
GO no.	Gene ontology term	Corrected p-value	Male or female biased	Pathway ID	Pathway	Q-value	Male or female biased
Cellular component				ko03015	mRNA surveillance pathway	1.85E-14	Male biased
GO:0005665	DNA-directed RNA polymerase II, core complex	2.59E-05	Male biased	ko03013	RNA transport	1.87E-08	Male biased
GO:0000428	DNA-directed RNA polymerase complex	0.00051	Male biased	ko00534	Glycosaminoglycan biosynthesis - heparan sulfate	4.74E-03	Male biased
GO:0030880	RNA polymerase complex	0.00051	Male biased	ko02010	ABC transporters	1.32E-02	Male biased
GO:0055029	Nuclear DNA-directed RNA polymerase complex	0.00051	Male biased	ko00900	Terpenoid backbone biosynthesis	3.49E-02	Male biased
GO:0005665	DNA-directed RNA polymerase II, core complex	2.59E-05	Male biased	ko00190	Oxidative phosphorylation	6.77E-26	Female biased
GO:0005739	Mitochondrion	2.70E-32	Female biased	ko00100	Steroid biosynthesis	4.43E-17	Female biased
GO:0044429	Mitochondrial part	6.55E-28	Female biased	ko01100	Metabolic pathways	2.36E-13	Female biased
GO:0044455	Mitochondrial membrane part	1.14E-17	Female biased	ko00970	Aminoacyl-tRNA biosynthesis	1.31E-11	Female biased
GO:0000313	Organellar ribosome	2.49E-17	Female biased	ko03030	DNA replication	1.64E-04	Female biased
GO:0005761	Mitochondrial ribosome	2.49E-17	Female biased	ko04350	TGF-beta signaling pathway	2.98E-04	Female biased
GO:0005746	Mitochondrial respiratory chain	5.51E-17	Female biased	ko04962	Vasopressin-regulated water reabsorption	9.56E-04	Female biased
GO:0005840	Ribosome	8.15E-17	Female biased	ko00020	Citrate cycle (TCA cycle)	9.56E-04	Female biased
Molecular function				ko04080	Neuroactive ligand-receptor interaction	3.11E-03	Female biased
GO:0003899	DNA-directed RNA polymerase activity	2.30499E-05	Male biased	ko04110	Cell cycle	1.23E-02	Female biased
GO:0034062	RNA polymerase activity	2.30499E-05	Male biased	ko03420	Nucleotide excision repair	1.81E-02	Female biased
GO:0003824	Catalytic activity	3.27116E-05	Male biased	ko03008	Ribosome biogenesis in eukaryotes	1.98E-02	Female biased
GO:0019003	GDP binding	0.00072	Male biased	ko00630	Glyoxylate and dicarboxylate metabolism	2.28E-02	Female biased
GO:0004631	Phosphomevalonate kinase activity	0.00119	Male biased	ko03010	Ribosome	2.37E-02	Female biased
GO:0004812	Aminoacyl-tRNA ligase activity	1.13E-16	Female biased	ko04964	Proximal tubule bicarbonate reclamation	3.19E-02	Female biased
GO:0016875	Ligase activity, forming carbon-oxygen bonds	2.12E-16	Female biased				
GO:0016876	Ligase activity, forming aminoacyl-tRNA and related compounds	2.12E-16	Female biased				
GO:0003954	NADH dehydrogenase activity	8.17E-15	Female biased				
GO:0003735	Structural constituent of ribosome	1.47E-12	Female biased				
Biological process							
GO:0008299	Isoprenoid biosynthetic process	3.68E-06	Male biased				
GO:0006720	Isoprenoid metabolic process	9.90E-05	Male biased				
GO:0021953	Central nervous system neuron differentiation	0.00023	Male biased				
GO:0009240	Isopentenyl diphosphate biosynthetic process	0.00033	Male biased				
GO:0019287	Isopentenyl diphosphate biosynthetic process, mevalonate pathway	0.00033	Male biased				
GO:0006412	Translation	9.80E-29	Female biased				
GO:0044249	Cellular biosynthetic process	5.81E-21	Female biased				
GO:1901576	Organic substance biosynthetic process	2.21E-20	Female biased				
GO:0009058	Biosynthetic process	9.88E-19	Female biased				
GO:0044710	Single-organism metabolic process	6.71E-18	Female biased				

Enriched pathways (Table 4) associated with male-biased DEGs were mRNA surveillance pathway, RNA transport, ABC transporters, terpenoid backbone biosynthesis etc. And enriched pathways for female-biased DEGs were oxidative phosphorylation, metabolic pathways, aminoacyl-tRNA biosynthesis, steroid biosynthesis, ribosome biogenesis in eukaryotes, DNA replication, vasopressin-regulated water re-absorption, neuroactive ligand-receptor interaction, cell cycle, nucleotide excision repair etc.

According to the enrichment analysis results, transcription was the most enriched activity in testis and translation and mitochondrion synthesis were the most enriched activities in ovary. Further studies on DEGs associated with the GO terms and pathways are needed to reveal the different molecular mechanisms that apparently exist between ovarian and testicular developmental processes.

### Real-time PCR confirmation of sex-biased genes

**Fig. 4:**
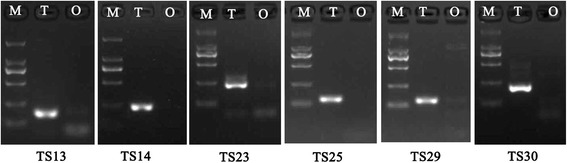
Testis-specific genes. M, DNA marker DL2000; T, testis; O, ovary. TS are the series numbers for testis-specific genes from bioinformatics analysis

**Fig. 5:**
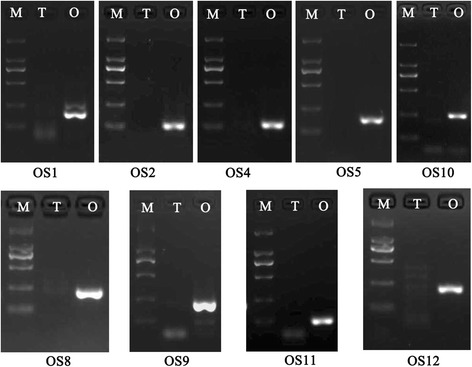
Ovary-specific genes. M, DNA marker DL2000; T, testis; O, ovary. OS are the series numbers for ovary-specific genes from bioinformatics analysis

One hundred and four DEGs with different levels of FPKM and FDR values were selected for RT-PCR validation to determine the veracity of the transcriptomic analysis. In male-biased genes, 6 of 14 testis-specific genes showed a specific testicular expression pattern by semi-quantitative PCR (Fig. 4), and the other 8 genes were significantly up-regulated in the testis as assessed with quantitative real-time PCR (Table 5). For the 33 male-biased DEGs, 32 showed the significantly up-regulated expression in testis, and only one gene was up-regulated in ovary (Table 5). In the female-biased genes selected, 9 of 14 PreO-specific genes in the ovary showed a specific expression pattern by semi-quantitative PCR (Fig. 5), and the other 5 genes were significantly up-regulated in ovary (Table 6). For the 43 female-biased DEGs, 33 showed up-regulated ovarian expression, 5 showed up-regulated testicular expression and 5 did not show expression differences between ovary and testis (Table 6). In total, 9 ovary-specific, 6 testis-specific, 45 testicular up-regulated and 39 ovarian up-regulated unigenes were obtained by real-time PCR confirmation.

**Table 5:** Summary of RT-PCR results for male-biased DEGs

Sequence ID	Bioinformatic analysis	Fold change	Semi-quantitative PCR validation	RQ (T/O)	qPCR validation (*T*-Test)	Annotation
Unigene916_All	TB	9.65	/	18.28	**	--
Unigene13080_All	TB	4.06	/	7.56	**	Solute carrier organic anion transporter family member 4C1
Unigene8661_All	TB	11.95	/	97.07	**	--
Unigene1205_All	TB	2.20	/	2.44	**	Neuron navigator 2
Unigene14488_All	TB	8.11	/	58.95	**	--
Unigene8480_All	TB	13.20	/	53.21	**	Transforming growth factor-beta-induced protein ig-h3
Unigene6291_All	TB	46.10	/	64.34	**	Receptor-type tyrosine-protein phosphatase T
Unigene2551_All	TB	11.69	/	59.28	**	Melanization interacting? protein
Unigene19396_All	TB	24.32	/	200.74	**	--
Unigene2838_All	TB	5.53	/	21.29	**	Intracellular fatty acid binding protein
CL911.Contig2_All	TB	47.53	/	342.15	**	--
Unigene6879_All	TB	56.75	/	84.84	**	Scylla paramamosain amino acids transporter
Unigene1920_All	TB	34.02	/	186.80	**	PREDICTED: 4-coumarate--CoA ligase-like
Unigene6062_All	TB	79.59	/	194.74	**	--
Unigene511_All	TB	25.83	/	102.59	**	Beta,beta-carotene 15,15′-monooxygenase
Unigene15520_All	TB	12.40	/	67.25	**	Cytochrome c oxidase subunit III
Unigene9139_All	TB	11.49	/	196.53	**	NADH dehydrogenase subunit 4
Unigene5297_All	TB	64.72	/	248.07	**	PREDICTED: metallothionein-1 F-like
Unigene6055_All	TB	11.66	/	56.41	**	Neuroparsin 1 precursor
CL756.Contig1_All	TB	16.05	/	19.52	**	Heme-binding protein 2
Unigene11959_All	TB	948.78	/	1415.06	**	--
CL550.Contig2_All	TB	27.87	/	−5.60	**	Alpha-I tubulin
Unigene15830_All	TB	825.49	/	3967.48	**	Histone H1-beta, late embryonic
Unigene7663_All	TB	22.26	/	54.81	**	PREDICTED: DNA excision repair protein haywire
Unigene16117_All	TB	4618.69	/	64216.17	**	Argonaute-3
CL2601.Contig2_All	TB	11.15	/	28.15	**	Homo sapiens X BAC RP11-1051 N9
CL535.Contig2_All	TB	133.51	/	33.55	**	Histone-lysine N-methyltransferase PRDM9
Unigene11824_All	TB	9.23	/	12.89	**	Ankyrin repeat domain-containing protein 13B
CL2710.Contig1_All	TB	10.35	/	13.38	**	Zinc finger protein on ecdysone puffs
Unigene3364_All	TB	5.50	/	3.10	**	Iodide transporter-like protein
Unigene46_All	TB	12.33	/	6.09	**	DNA ligase 4
CL730.Contig2_All	TB	10.32	/	19.81	**	PREDICTED: HIG1 domain family member 1C
Unigene15329_All	TB	18.22	/	21.07	**	Stromal antigen-like protein, copy A
Unigene15690_All	TB	3.78	/	8994.17	**	--
Unigene15762_All	TB	4.57	/	371865.46	**	--
Unigene20560_All	TB	114.97	/	1014.87	**	--
CL951.Contig2_All	TS	/	Not specific??	947.72	**	--
CL1031.Contig1_All	TS	/	Not specific	19779.35	**	Probable serine/threonine-protein kinase fhkB
CL273.Contig1_All	TS	/	Not specific	39469.77	**	Eukaryotic translation initiation factor 4 gamma, putative
Unigene1966_All	TS	/	Not specific	35.96	**	Tyrosine aminotransferase
Unigene16106_All	TS	/	Not specific	148.83	**	--
Unigene23134_All	TS	/	Specific	/	/	--
Unigene29005_All	TS	/	Specific	/	/	Casein kinase I
Unigene29287_All	TS	/	Specific	/	/	DNA-directed RNA polymerase II subunit RPB2
Unigene28792_All	TS	/	Specific	/	/	--
Unigene28151_All	TS	/	Specific	/	/	Dual specificity testis-specific protein kinase 2
CL737.Contig2_All	TS	/	Specific	/	/	Penaeus monodon clone TUZX4-6:48 microsatellite sequence

*TB* testis biased, *TS* testis specific, *RQ* relative expression level

Asterisks indicate significant difference between ovary and testis expression of the gene by QPCR test ( *P<0.05, **P<0.01)

**Table 6:** Summary of RT-PCR results for female-biased DEGs

Sequence ID	Bioinformatic analysis	Fold change	Semi-quantitative PCR validation	RQ (O/T)	qPCR validation (*T*-test)	Annotation
Unigene15643_All	OB	2.05	/	−2.32	**	SUMO-activating enzyme subunit 1
Unigene13333_All	OB	40.71	/	−1.75	N	Hypothetical protein
Unigene13199_All	OB	13.27	/	−1.72	*	Facilitated trehalose transporter Tret1
Unigene4124_All	OB	2.2	/	−1.62	**	DNA ligase 1
Unigene3159_All	OB	8.44	/	−1.44	**	--
Unigene5760_All	OB	4.17	/	−1.32	N	Tail muscle elongation factor 1 gamma
Unigene9807_All	OB	52.44	/	−1.3	*	--
CL1556.Contig1_All	OB	6.53	/	−1.11	N	Hemolymph clottable protein
Unigene4414_All	OB	3.61	/	−1.01	N	DNA replication complex GINS protein
Unigene13906_All	OB	2.81	/	1.12	N	NADH dehydrogenase [ubiquinone] 1 alpha subcomplex subunit 8
Unigene12464_All	OB	3.49	/	1.37	**	Vitelline membrane protein
CL1780.Contig2_All	OB	4.55	/	1.76	**	Cytosolic MnSOD
Unigene4283_All	OB	6.66	/	1.8	**	Ubiquitin carboxyl-terminal hydrolase isozyme L5
Unigene5769_All	OB	5.67	/	2.17	**	Mannose-P-dolichol utilization defect 1 protein homolog
Unigene2752_All	OB	3.21	/	2.83	**	39S ribosomal protein L27, mitochondrial
CL1767.Contig3_All	OB	36.82	/	3.76	**	Vitellogenin
Unigene10823_All	OB	3.77	/	4.49	**	GJ22360 [Drosophila virilis]
Unigene7488_All	OB	10.38	/	6	**	Megaderma lyra mitochondrial aldehyde dehydrogenase 2 (Aldh2) gene
CL745.Contig1_All	OB	73.15	/	10.4	**	JHE-like carboxylesterase 1
CL1422.Contig1_All	OB	35.44	/	21.85	**	Penaeus (Litopenaeus) vannamei microsatellite TUMXLv8.67 sequence
CL355.Contig1_All	OB	41.48	/	23.46	**	Metallothionein
Unigene12371_All	OB	67.42	/	30.75	**	Serine protease-like protein
Unigene2772_All	OB	129.19	/	52.27	**	Crustacyanin-A2 subunit
Unigene10728_All	OB	187.39	/	57.32	**	Putative microtubule-associated protein
CL160.Contig1_All	OB	274.18	/	58.75	**	Mucin-19
Unigene9289_All	OB	188.85	/	62.16	**	ATP binding cassette transmembrane transporter
CL1757.Contig1_All	OB	6.5	/	68.51	**	Pol-like protein
Unigene944_All	OB	232.28	/	78.45	**	--
Unigene2582_All	OB	82.88	/	83.62	**	Peroxidase
Unigene15565_All	OB	561	/	165.67	**	PREDICTED: hypothetical protein
Unigene15673_All	OB	230.04	/	180.28	**	Hypothetical protein UY3_06274
CL1544.Contig3_All	OB	2.35	/	279.59	**	Toll protein
Unigene7394_All	OB	186.3	/	309.91	**	Penaeus monodon progestin membrane receptor component 1 (PGMRC1)
CL92.Contig2_All	OB	99.85	/	743.69	**	Penaeus monodon polehole-like protein Mrna
Unigene10229_All	OB	64.19	/	754.82	**	--
Unigene11618_All	OB	1070.04	/	938.62	**	--
CL92.Contig3_All	OB	103.95	/	1017.81	**	Polehole-like protein
Unigene2102_All	OB	695.22	/	2497.98	**	Vitellogenin receptor
CL989.Contig2_All	OB	992.51	/	3232.49	**	Thrombospondin protein
CL2390.Contig2_All	OB	226.25	/	3345.08	**	Cyclin B
Unigene9017_All	OB	215.42	/	3401.83	**	Cyclin B
CL2487.Contig2_All	OB	1015.41	/	7095.22	**	Thrombospondin
CL1646.Contig1_All	OB	1025.74	/	11552.92	**	Thrombospondin-type laminin G domain and EAR repeat-containing protein
Unigene905_All	OS	/	Not specific	6.07	**	Zebrafish DNA sequence from clone CH211-150 K4 in linkage group 1
CL496.Contig2_All	OS	/	Not specific	23.23	**	Glutamate receptor ionotropic, NMDA 3A
Unigene7304_All	OS	/	Not specific	72.23	**	X-linked retinitis pigmentosa GTPase regulator [Tupaia chinensis]
Unigene12972_All	OS	/	Not specific	117.13	**	Peroxidasin
CL1169.Contig2_All	OS	/	Not specific	315.15	**	PREDICTED: uncharacterized protein
Unigene12309_All	OS	/	Specific	/	/	Dual oxidase maturation factor 1
Unigene8249_All	OS	/	Specific	/	/	PREDICTED: guanine nucleotide-binding protein G(q) subunit alpha-like
Unigene21442_All	OS	/	Specific	/	/	--
Unigene9242_All	OS	/	Specific	/	/	PREDICTED: MD-2-related lipid-recognition protein-like
Unigene1049_All	OS	/	Specific	/	/	Amyloid beta A4 protein
CL57.Contig2_All	OS	/	Specific	/	/	Gamma-interferon-inducible lysosomal thiol reductase
CL711.Contig1_All	OS	/	Specific	/	/	Mucin-5 AC (Fragments)
Unigene4288_All	OS	/	Specific	/	/	--
Unigene859_All	OS	/	Specific	/	/	--

*OB* ovary biased, *OS* ovary specific, *RQ* relative expression level

Asterisks indicate significant difference between ovary and testis expression of the gene by QPCR test ( *P<0.05, **P<0.01)

**Fig. 6:**
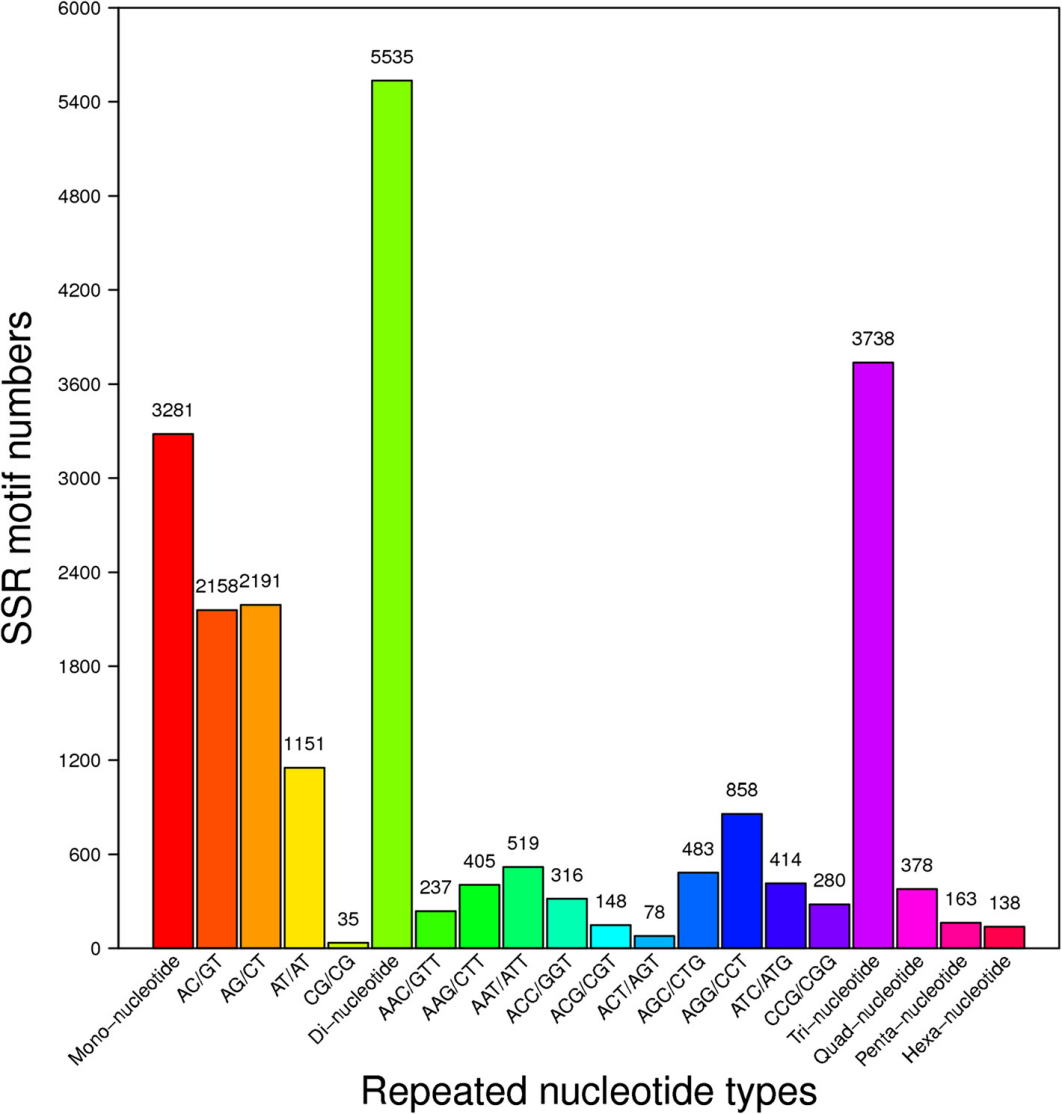
Distribution of putative SSRs in the transcriptome of the *L. vannamei* gonad. X-axis, distribution of SSR types; Y-axis, number of different SSR types

SSRs in the gonadal transcriptome of* L. vannamei* SSR detection was performed with the MicroSAtellite (MISA) software using all-unigenes as references. A total of 13,233 SSRs were identified in 10,411 unigene sequences, with 2,192 (21.05 %) unigenes containing more than one SSR. Among the different types of SSRs, the di-nucleotide repeats were the most abundant, accounting for 41.83 %, followed by the tri-nucleotide repeats (28.25 %), mono-nucleotide repeats (24.79 %), quad-nucleotide repeats (2.86 %), penta-nucleotide repeats (1.23 %) and hexa-nucleotide repeats (1.04 %) (Fig. 6). The distributions of SSRs in unigenes and primers for SSRs are shown in Additional file 1: Table S6.

## Discussion

*L. vannamei* is one of the most important aquaculture species and has attracted the attention of many researchers. A high-throughput RNA sequencing strategy has already been used in studying the mechanisms of viral resistance [50–53] and nitrite adaptation [54], as well as in revealing gene expression patterns in muscle, hepatopancreas, gills and pleopods of normal shrimp [55, 56]. However, data regarding its reproductive or sex phenotypes is lacking. Herein, we performed RNA-Seq on the gonads of adult males and females in an attempt to unravel sex-related genes. The reason we chose adult individuals for gonadal transcriptomic analysis was that we focused primarily on genes involved in gonadal development and gametogenesis. Moreover, these cell types showed more diversity in adult gonads; for example, oogonia/spermatogonia, and primary and secondary oocytes/spermatocytes are all present in adult gonads (Additional file 1: Figure S1). This stage selection was appropriate since we identified in our assembled sequences a great majority of genes involved in gonadal development, oogenesis, and spermatogenesis, as well as some sex-determining genes previously reported.

### Reference gonadal transcriptome of *L. vannamei*

*De-novo* assembly of the sequencing data from four *L. vannamei* gonadal cDNA libraries resulted in 65,218 unigenes with an average length of 1021 bp. Approximately half of the unigenes (46.47 %) had significant matches against existing sequences and 40.65 % were annotated using Gene Ontology terms. The *L. vannamei* sequences possessed top matches with *D. pulex* sequences, since this latter species is also a crustacean and its entire genome has been sequenced [57]. KEGG pathway analysis showed that, the main biological pathways involved in gonadal development and gametogenesis were obtained, which will facilitate the further in-depth analysis of the relationships between different genes in the transcriptome of gonads. However, there were far fewer pathways involved in spermatogenesis than in oogenesis, and more female-biased genes (75.6 %) than male-biased genes (37.9 %) showed NR hits. This could be due to the fact that researchers have mainly focused on the reproductive phenotypes of female crustaceans because of their economic importance to aquaculture [15, 26]; thus, genetic information for female crustaceans has been much richer than for males.

### Sex-determining genes

The transcription factor, sex-determining region of the Y-chromosome (*Sry*), is the sex-determining gene in mammalians. The correct expression of *Sry* triggers testicular development, while decreased or delayed *Sry* expression leads to testicular defects [58]. *Sry* acts by activating the *Sox9* gene [59], and then, downstream genes of *Sox9* promote testis development. Once expression of *sox9* or its downstream genes is impeded, the gonad can switch to ovarian development [60]. Doublesex (*Dsx*) is a male sex-determining gene that plays an important role in controlling sexual dimorphism in invertebrate organisms such as nematodes, insects and *Daphnia magna,* as well as in vertebrates [61–63]. *Fem-1* is also a male sex-determining gene required for somatic and germline development in the testis of *C. elegans* [64, 65]. Herein, we identified all male sex-determining genes mentioned above in *L. vannamei*, including a testicular up-regulated *Sry* (Unigene7231_All), sox9 (CL86.Contig2_All, Unigene2458_All), *Dsx* (Unigene35364_All) and seven *fem-1* or *fem-1-like* genes (Unigene24042_All, Unigene9434_Al, Unigene9463_All, Unigene24047_All, Unigene11407_All, Unigene2036_All, Unigene15025_All).

*Foxl2* (Forkhead box l2) encodes a conserved transcription factor and is a sex determiner in female vertebrates. It is preferentially expressed in the ovary and involved in ovarian differentiation and maintenance by repression of testis-specific genes [66, 67]. Although *foxl2* orthologs have been cloned in some invertebrates, its role in sex determination or differentiation remains unclear [68]. We found *foxl2* (Unigene24037_All) in our gonadal transcriptomic data, and intriguingly, it shows up-regulated expression in testis.

The identification of known sex-determining genes in the present study proved that transcriptomic sequencing is a powerful tool in mining sex-determining genes. The exact role of these genes in sex determination of *L. vannamei* remains to be examined.

### Identification of genes involved in germline determination and development

Gonadal tissue is composed of germ cells and somatic cells. Germ cells are the only cells that can undergo both meiosis and mitosis, and finally differentiate into gametes (spermatozoon and oocyte); and sperm-oocyte binding in sexually reproducing organisms constitutes the beginning of the next generation. To maintain germ cell fate and regulate its development and differentiation, gene expression patterns in germ cells need to be regulated at the transcriptional and post-transcriptional levels. Moreover, it is also important to maintain the niche formed by somatic cells so as to maintain the characteristics of germ cells. Transcriptional regulation of gene expression is widely used in early differentiation stages of germ cells. In mouse, interaction of *blimp1* (*B-lymphocyte-induced maturation protein 1*) with *Prmt* (*histone methyltransferase*) represses chromatin transcription and prevents trans-differentiation from germ cells to somatic cells. In the blimp1-knockout mouse, germ cells cannot form [69–72]. In the present study, a *blimp1* (Unigene15201_All), three *Prmt* (*PRMT2*: Unigene7507_All, *PRMT3*: CL2301.Contig2_All, CL2301.Contig1_All, *PRMT6*: Unigene14757_All), and a chromatin target of Prmt1 protein genes (Unigene4147_All) were identified, indicating a similar regulation of germ cell fate at the transcriptional level in shrimp.

The regulation of RNA translation, however, is more common and important in germ cells. A battery of evolutionally conserved RNAs is essential for germ cell proliferation, survival and differentiation; some of these genes are called germ cell markers, because they exist throughout the germ cell life cycle. *Vasa*, first isolated in *Drosophila*, is expressed throughout germ cell developmental stages in many invertebrates and vertebrates species [73, 74]. As a molecular marker, *vasa* was used to track germ cell specification, migration and differentiation [75–77]; and loss of function of vasa led to germ cell specification failure and defects in germ cell development in *Drosophila*, mouse and nematode [78–81]. *Vasa* were hypothesized to function by inhibiting expression of genes that are responsible for somatic differentiation in germ cells [82]. This hypothesis was further supported by studies of *nanos*, a target of *vasa*, which can act together with *pumilio* to repress mitosis, transcription and translation during the development of the germline [83–85]. The DAZ (*Deleted in Azoospermia*) family is another well-known gene family that consists of three genes specifically expressed in germ cells: *BOULE*, *DAZ-Like* (*DAZL*) and *DAZ. DAZ* and *DAZL* are expressed throughout almost the entire life cycles of germ cells, and are essential for germ cell determination, differentiation and maturation; while *BOULE* is mainly involved in germ cell meiosis [86–89]. In the present study, *vasa* (Unigene1169_All), *nanos* (Unigene22266_All), *pumilio* (CL285.Contig16_All), *boule* (Unigene1947_All) and three *DAZ-associated protein* (*daz interacting protein* 1, Unigene13632_All; DAZ-associated protein 1-like, Unigene20_All; *DAZ-associated protein 2*, Unigene4428_All) were identified, offering abundant genetic information for further study of germline determination and development.

miRNAs are also involved in RNA regulation of germ cells. Knockout of the *Dicer* gene prevented splicing of miRNAs--the regulator of germ cell-specific genes-- leading to the termination of germ cell development [90]. Additionally, the PIWI-mediated piRNA pathway regulates the expression of germ cell determination genes, such as *vasa* and *oskar*. The PIWI-null mutant fly (*Drosophila*) and mouse exhibited a deficiency in germ cell formation and differentiation [91, 92]. The existence of *Dicer* (Unigene17990_All) and two *piwi* (*piwi* 1, Unigene399_All; *piwi* 2, Unigene21063_All) in our transcriptome made miRNA-mediated regulation in *L. vannamei* possible.

The gonadal cell niche surrounding germ cells is also important for the preservation of germ cell fate [93]. For example, Notch signaling and its downstream genes in nematode [94], *BMPs* (bone morphogenetic proteins) in *Drosophila* [95] and *GDNF* (*glial cell line-derived neurotrophic factor*, *GDNF*) in mammals are produced by gonadal somatic cells [96] such as Sertoli cells, and are the known pathway or factors that might control meiotic timing and germ cell numbers. We retrieved two *notch* genes (*Notch*, Unigene14535_All; *Notch2*, Unigene14705_All); two *BMP* genes (BMP1, Unigene14770_All; BMP7, Unigene12011_All); and two *GDNF associated genes* (*GDNF-inducible zinc finger protein 1*, Unigene4392_All; *GDNF family receptor alpha-3*, Unigene7144_All) in the gonadal transcriptome of *L. vannamei*. Moreover, 101 sequences for genes in the Wnt signaling pathway were also obtained. This information will further benefit the study of the mechanisms of interaction between germ cells and somatic cells in *L. vannamei*.

### Sex-biased genes involved in spermatogenesis and oogenesis are identified

Except for similar cell lines in both male and female gonads, there are remarkable differences between the ovary and testis that involve morphology, cell types and biologic processes; and also gene expression patterns and molecular regulatory mechanisms. A large number of gonadal differentially expressed genes have been identified in the present transcriptome, and many more genes were over-expressed in the testis (19,279) compared to the ovary (3,529). This male-biased gene expression pattern in the gonads has been observed in *Drosophila* [97, 98], *Caenorhabditis elegans* [99], fishes [100, 101] and mammals [102], as well as in the green mud crab (*Scylla paramamosain*) [36], a crustacean species. This phenomenon may be explained by the previous assumption that male development is regulated by activating a series of testis-specific genes and/or by repressing genes vital for ovarian development [103, 104].

The most important biologic processes in the testis and ovary are spermatogenesis and oogenesis, respectively. As expected, testis-specific and differentially expressed genes include members with functions pertinent to spermatogenic stages. For example, the male-biased gene *spermatogonial stem-cell renewal factor* (CL2114.Contig2_All), *MLH1* (CL2662.Contig1_All, CL2662.Contig2_All) and *RHAU* (Unigene22544_All) are essential for early stages of spermatogenesis, including spermatogonial maintenance and differentiation; and knockout of *RHAU* or *MLH1* resulted in deficiencies in spermatogonial or spermatocytic differentiation in the mouse [105–107]. Genes involved in later stages of spermatogenesis include *Spermatogenesis-associated proteins* (*Spata2*, Unigene8309_All; *Spata5* (CL1808.Contig1_All, CL1808.Contig2_All); Spata20, Unigene9757_All), *spermatogenesis regulator* (Unigene14897_All); sperm associated antigens ciliary and flagellar proteins such as *Sperm-associated antigen* (*Spag2*, Unigene7744_All; *Spag7*, *Unigene482_All*); *Sperm-specific protein PHI-2B/PHI-3* (Unigene12515_All); *Sperm protamine P2* (Unigene20056_All); *Spermatid-specific protein T1* (Unigene12459_All, Unigene17251_All); *Round spermatid basic protein 1* (Unigene6458_AlL); *major sperm protein* (Unigene9914_All); and *Motile sperm domain-containing protein 2* (Unigene9914_All). In addition, the testis-biased genes *testis-specific serine proteases* (*TESSP*) (Unigene17312_All, Unigene2408_All, Unigene25019_All) are involved in germ cell survival during meiosis [108, 109], and *T-complex testis-specific protein 1*(*t-complex 11*) (Unigene4999_All) might function in insemination processes so as to stimulate sperm capacitation and inhibit the acrosome reaction [110].

Among the female-biased sequences, we focused on genes involved in oogenic processes. Vitellogenesis is the central process in oogenesis. In crustaceans, *vitellogenin*--the precursor of yolk protein--is synthesized in the hepatopancreas and ovary [111, 112]. The synthesis of *vitellogenin* in oviparous vertebrates is regulated by the E2-ER-Hsp90-Vtg pathway, in which estrogen receptor (ER) and heat shock protein 90 (Hsp90) mediate the enhancement of *vitellogenin* transcription by estrogen or estrogen-like hormones [113, 114]. In our transcriptomic data, we identified *vitellogenin* (CL1767.Contig3_All), *vitellogenin receptor* (*VgR*) (Unigene2102_All), *estrogen receptor* (CL2390.Contig2_All, Unigene9144_All), *estrogen receptor-binding protein* (Unigene2782_All) and *HSP90* (Unigene15703_All) genes, indicating that an E2-ER-Hsp90-Vtg pathway exists in crustaceans. Further experiments should focus on whether and how E2, ER and Hsp90 regulate the synthesis of *vitellogenin* in shrimp.

The second meiotic division and oocyte maturation constitute another vital process in oogenesis. MPF (maturation-promoting factor), mainly composed of CDK1 (encoded product of the *Cdc2* gene) and Cyclin B proteins, is a primary regulator of this process. MPF can be activated by G2 to reach the M phase transition, and the active MPF promotes rapid maturation of oocytes [115]. In the present study, we identified *G2/mitotic-specific cyclin-A* (Unigene10629_All), *cyclin-B* (Unigene4127_All, Unigene1805_All), *cyclin-B3* (CL352.Contig2_All), *cyclin-F* (Unigene11044_All); and the components of MPF, *Cdc2* (Unigene3813_All) and *Cyclin B* (CL2390.Contig3_All). The identification of these genes will facilitate further study on the artificial induction of oocyte maturation in *L. vannamei*. In addition, oocyte quality is hypothesized to be improved by high levels of the expression of superoxide dismutase genes, which could neutralize reactive oxygen species and protect the embryo during its development [116]. Herein, we found four superoxide dismutase homologs (Unigene14142_All, Unigene419_All, Unigene6388_All, Unigene8984_All) that are predominantly expressed in the ovary, indicating a similar maternal protection of offspring by shrimp; and antioxidant defense techniques may be useful in improving the overall quality of shrimp larvae.

### Verification of sex-biased genes

Among 104 differentially expressed gonadal unigenes, 9 genes specifically expressed in ovary and 6 specifically expressed in testis were verified by semi-quantitative RT-PCR; while of the remaining DEGs tested, 45 testis-predominant unigenes and 39 ovary-predominant unigenes were verified by quantitative real-time PCR analysis. These gonadal specific and differentially expressed genes must play important roles in certain parts of gonadal development in *L. vannamei*. However, there are some differences observed between transcriptomic analysis and qRT-PCR data concerning fold-changes in gene expression in testis and ovary, and one male-biased DEG showed ovarian up-regulated expression and 5 female-biased DEGs showed testis-predominant expression. This is most likely due to the differences that often existing between the bioinformatics analysis of next-generation sequencing technology and the actual experimental analysis [117]; and biologic replicates (which are more valuable and accurate for detecting differently expressed genes), were not available during the course of the present study [118].

### Discovery of SSR markers

Simple sequence repeats (SSRs) are single-locus markers with high allelic variation and are widely applicable to molecular genetics studies, including research involving genetic diversity assessment, comparative genomics, gene flow characterization, and genetic linkage mapping [119]. In *L.vannamei,* a number of microsatellite sequences have been reported for genetic map construction and quantitative trait locus (QTL) detection [120–122], and next-generation sequencing has facilitated the discovery of a relatively large set of SSRs in the hepatopancreas of the shrimp by the present team [53, 123]. The SSRs identified in this study can serve as genetic markers for quantifying genetic diversity of germplasm within breeding and imported populations, identifying paternity of the breeding families and managing broodstocks, which are the major issues for the selectively breeding and farming of introduced species. They can also be used in QTL mapping and marker-assisted select ion (MAS) in *L. vannamei* and other phylogenetically similar shrimp species. This may promote genetic gain to traits of interest for aquaculture, such as reproduction, sex determination, growth, and tolerance against environmental stress.

## Conclusions

The present study encompasses the first large-scale RNA sequencing of shrimp gonads. We have identified many important sex-related functional genes, GO terms and pathways, all of which will facilitate future research into the reproductive biology of shrimp. The SSRs detected in this study can be used as genetic markers for germplasm evaluation of breeding and imported populations.

## Additional file

Additional file 1: Figure S1. Histological features of the testis and pre-eyestalk ablation shrimp ovary. Both gonads are in early development stages and contain mostly spermatogonia, spermatocytes, oogonia and oocytes. **Table S1.** List of KEGG pathways for gonadally expressed genes. **Table S2.** List of differentially expressed genes in male and female gonads. (FDR < 0.001). **Table S3.** Enriched GO terms and pathways for sex-biased genes. **Table S4.** Primers and verification information of male-biased genes. **Table S5.** Primers and verification information of female-biased genes. **Table S6.** The distributions of SSRs in unigenes and primers for SSRs. (ZIP 3741 kb)

## Abbreviations

cDNA: complementary DNA
Day1O: ovary of one-day post-eyestalk ablation female shrimp
Day6O: ovary of six-day post-eyestalk ablation female shrimp
DEGs: differentially expressed genes
FDR: false discovery rate
FPKM: fragments per kilo base per million
GO: gene ontology
NR: NCBI non-redundant protein database
PreO: Ovary of pre-eyestalk ablation female shrimp
